# CCL28 Induces Mucosal Homing of HIV-1-Specific IgA-Secreting Plasma Cells in Mice Immunized with HIV-1 Virus-Like Particles

**DOI:** 10.1371/journal.pone.0026979

**Published:** 2011-10-31

**Authors:** Veronica Rainone, Gregor Dubois, Vladimir Temchura, Klaus Überla, Alberto Clivio, Manuela Nebuloni, Eleonora Lauri, Daria Trabattoni, Francisco Veas, Mario Clerici

**Affiliations:** 1 Department of Clinical Sciences, University of Milan, Milan, Italy; 2 UMR-MD3, Comparative Molecular Immuno-Physiopathology Laboratory, Faculty of Pharmacy, University of Montpellier, Montpellier, France; 3 Department of Molecular and Medical Virology, Ruhr-University Bochum, Bochum, Germany; 4 FoldLESs S.r.l., Department of Clinical Sciences, University of Milan, Milan, Italy; 5 Department of Biomedical Sciences and Technologies, University of Milan, Milan, Italy; 6 Don C. Gnocchi Foundation, IRCCS, Milan, Italy; University of Alabama, United States of America

## Abstract

Mucosae-associated epithelial chemokine (MEC or CCL28) binds to CCR3 and CCR10 and recruits IgA-secreting plasma cells (IgA-ASCs) in the mucosal *lamina propria.* The ability of this chemokine to enhance migration of IgA-ASCs to mucosal sites was assessed in a mouse immunization model using HIV-1_IIIB_ Virus-like particles (VLPs). Mice receiving either HIV-1_IIIB_ VLPs alone, CCL28 alone, or the irrelevant CCL19 chemokine were used as controls. Results showed a significantly increased CCR3 and CCR10 expression on CD19^+^ splenocytes of HIV-1_IIIB_ VPL-CCL28-treated mice. HIV-1 Env-specific IFN-γ, IL-4 and IL-5 production, total IgA, anti-Env IgA as well as gastro-intestinal mucosal IgA-secreting plasma cells were also significantly augmented in these mice. Notably, sera and vaginal secretions from HIV-1_IIIB_ VLP-CCL28-treated mice exhibited an enhanced neutralizing activity against both a HIV-1/B-subtype laboratory strain and a heterologous HIV-1/C-subtype primary isolate. These data suggest that CCL28 could be useful in enhancing the IgA immune response that will likely play a pivotal role in prophylactic HIV vaccines.

## Introduction

The development of prophylactic and therapeutic vaccines against mucosal infections still represents a challenge [1], [2]. IgA secretion form mucosal tissues constitutes the first and main adaptive immunity line of defence against such infections [3], [4], [5], thus, it is conceivable that efficient prophylactic vaccines for these infections should elicit the production of IgA. IgA-secreting plasma blasts and plasma cells (IgA-ASCs) are characterized by a number of surface receptor proteins, including CCR3 and CCR10, which are bound by specific chemokines [6], [7]. In particular, both CCR3 and CCR10 bind CCL28, a CC chemokine also known as Mucosae-associated Epithelial Chemokine, or MEC [6], [8], [9], [10]. These interactions are involved in both migration and recruitment of IgA-ASCs into mucosal *lamina propria* (MLP) [4], [10], [11]. CCL28 is widely expressed in epithelial tissues of various mucosal sites and it chemoattracts IgA-ASCs originated from mucosal lymphoid organs in mucosal effector sites, including the large and small intestine, bronchi, the mammary as well as salivary glands in both mice and humans [4].

We recently reported a positive correlation between titers of mucosal anti-HIV-1 IgA and the CCL28–CCR3/CCR10 system both in HIV-1 infected and HIV-1-exposed but sero-negative (HESN) individuals [12]. Furthermore, we demonstrated that the recruitment of IgA-ASCs in the *lamina propria* is enhanced in mice immunized with Vesicular Stomatitis Virus (VSV) in the presence of *in vivo* CCL28 [12]. These observations could be important in suggesting how to optimally build a mucosal vaccine for the prevention of HIV infection. In this context, Virus-like Particles (VLPs) represent a novel vaccine approach based on non-pathogenic particles mimicking the size and the structure of the cognate viruses [13], [14], [15], [16]. Notably, VLPs were shown to elicit broad immune responses in animal models and to stimulate cell and humoral immune responses *via* the MHC class I and class II antigen presentation pathways [17], [18], [19].

Since mucosal vaccines aim at inducing antigen-specific secretory IgA antibodies in addition to effective cell-mediated responses, the use of chemokines as adjuvants could significantly improve vaccine immunogenicity and efficacy [20], [21]. Chemokines and their corresponding receptors are indeed the main effector molecules of the common mucosal immune system and are essential for regulating the homing of immune-competent cells from the site where immune responses are induced to the mucosal effector sites [3]. In this respect, CCL28 could represent a suitable candidate as mucosal molecular adjuvant. Notably, the usefulness of CCL28 as an adjuvant for mucosal vaccination was confirmed by recent data showing that the association of CCL28 and a DNA-based vaccine results in the generation of a potent immune response that prevents mucosally transmitted viral infections [22].

In the present study, we further investigate the immunomodulatory effects of MEC/CCL28 in a mouse model using a prime-boost strategy based on HIV-1_IIIB_-VLPs in the presence/absence of the murine chemokine gene inserted into a CpG-free expression vector. Results confirm the pivotal role of CCL28 in the modulation of mucosal immunity and suggest that CCL28 could be useful in the design of mucosal vaccines finalized at the prevention of HIV infection.

## Results

### *In vivo* expression of CCL28 and CCL19

**Figure 1:**
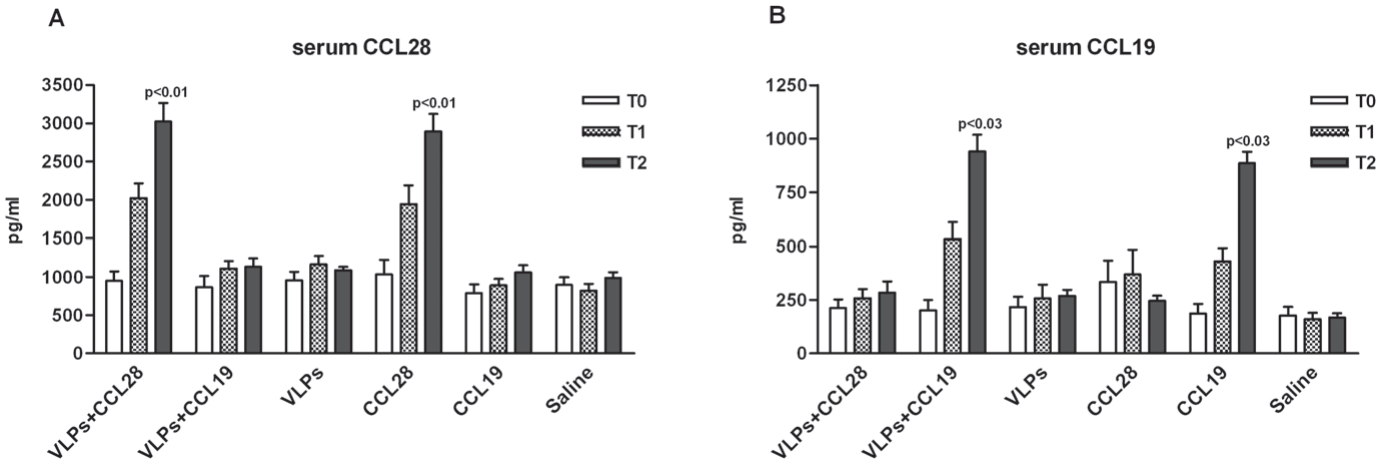
*In vivo* expression of recombinant CCL28 and CCL19. Panels *A* and *B* respectively represent concentrations (pg/ml) of CCL28 and CCL19 in sera from each mice group immunized with: HIV-1-VLP_IIIB_-CCL28, HIV-1-VLP_IIIB_-CCL19, HIV-1-VLP_IIIB_, CCL28 alone, CCL19 alone and control (saline) at days 0 (T0), 14 (T1) and 28 (T2). The data are expressed as mean ± SD (n = 5 mice per group) of three independent experiments. Statistically significant differences are shown.

Inbred female Balb/c mice were immunized with a prime-boost regime based on HIV-1_IIIB_ VLP in the presence or in the absence of CCL28-expressing vector. The CCL19-expressing plasmid was used as a negative control. This chemokine was chosen because, similarly to CCL28, it plays a crucial role in lymphoid cell trafficking but nevertheless it does not have an effect on IgA^+^ plasma cells; additionally, CCL19 does not bind CCR3 or CCR10 but rather uses CCR7. To verify if pCCL28 and pCCL19 were effective expression vectors and whether their use resulted in systemic increases of the corresponding chemokines, CCL28 an CCL19 were measured in immune sera. Results showed a significant increase in serum CCL28 levels of HIV-VLP_IIIB_-CCL28 and CCL28 alone mice compared to HIV-VLP_IIIB_-CCL19, HIV-VLP_IIIB_, CCL19 alone and saline mice (p<0.01 in all cases). Similar results were observed when serum CCL19 levels from both HIV-VLP_IIIB_-CCL19 and CCL19 alone mice were compared to those obtained in HIV-VLP_IIIB_-CCL28, HIV-VLP_IIIB_, CCL28 alone or saline animals (p<0.03 in all cases) (Fig. 1).

### CCR3- and CCR10- expressing CD19^+^ cells

**Figure 2:**
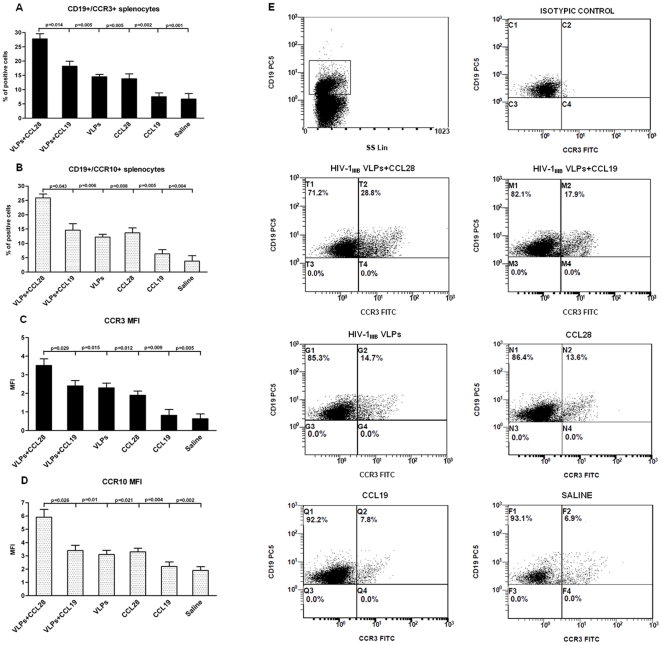
Analysis of CCR3 and CCR10 receptor expression on CD19^+^ B splenocytes. Percentage of CCR3-expressing CD19^+^ splenocytes (*A*), percentage of CCR10-expressing CD19^+^ splenocytes (*B*). Flow cytometry analysis of expression of the CCR3 receptor (*C*) or CCR10 receptor (*D*) at the surface of CD19^+^ splenocytes (MFI). Data represents three different lots of independent experiments. Mean values ± SD and statistically significant differences between each immunized mice group and the reference HIV-1-VLP_IIIB_-CCL28 group are indicated. CCR3 expression on B-cells examined by flow cytometry (*E*). For each analysis, 20000 events were acquired and gated on CD19 expression and side scatter properties. The cells were stained with anti-CCR3 monoclonal antibody, as described in Materials and Methods. The percentages of CCR3^+^ cells are indicated in Results. The data are from a single representative experiment of three similar experiments performed for freshly isolated CD19^+^ splenocytes.

To analyze the expression of chemokine receptors on circulating lymphocytes, CCR3- and CCR10-expressing CD3^+^, CD14^+^ and CD19^+^ splenocytes were analyzed in all mice. Results showed that CD19^+^/CCR3^+^ and CD19^+^/CCR10^+^ splenocytes were significantly augmented in HIV-VLP_IIIB_-CCL28 treated mice as compared to HIV-VLP_IIIB_-CCL19 (p = 0.014 and 0.043, respectively), HIV-VLP_IIIB_ alone (p = 0.005 and 0.006, respectively), CCL28 alone (p = 0.005 and 0.008, respectively), CCL19 alone (p = 0.002 and 0.005, respectively), and saline control mice (p = 0.001 and 0.004, respectively) (Fig. 2A and 2B). CD19^+^/CCR3^+^ and CD19^+^/CCR10^+^ cells were also augmented, although not in a statistically significant manner, in both HIV-VLP_IIIB_-CCL19 and HIV-VLP_IIIB_ alone groups of animals as compared to control groups. However, these results were not completely unexpected, because the use of VLP-based vaccine strategies have been shown to effectively activate both arms of the immune response and a direct and/or an indirect effect on lymphocyte phenotype cannot be excluded. In contrast with these results, CCR3 and CCR10 expression was comparable in CD3^+^ splenocytes and CD14^+^ cells of all groups (data not shown).

### Surface density of CCR3 and CCR10 on CD19^+^ cells

Evaluation of mean fluorescence intensity (MFI) showed that the CCR3 MFI on CD19^+^ splenocytes was significantly augmented in HIV-VLP_IIIB_-CCL28 treated mice compared to HIV-VLP_IIIB_-CCL19 (p = 0.029), HIV-VLP_IIIB_ alone (p = 0.015), CCL28 alone (p = 0.012), CCL19 alone (p = 0.009), or saline control mice (p = 0.005) (Fig. 2C). CCR10 MFI on CD19^+^ lymphocytes was similarly upregulated in HIV-VLP_IIIB_-CCL28 treated mice compared to all the other groups (Fig. 2D).

### Cytokine production by HIV-1/Env-stimulated splenic and colon cells

**Figure 3:**
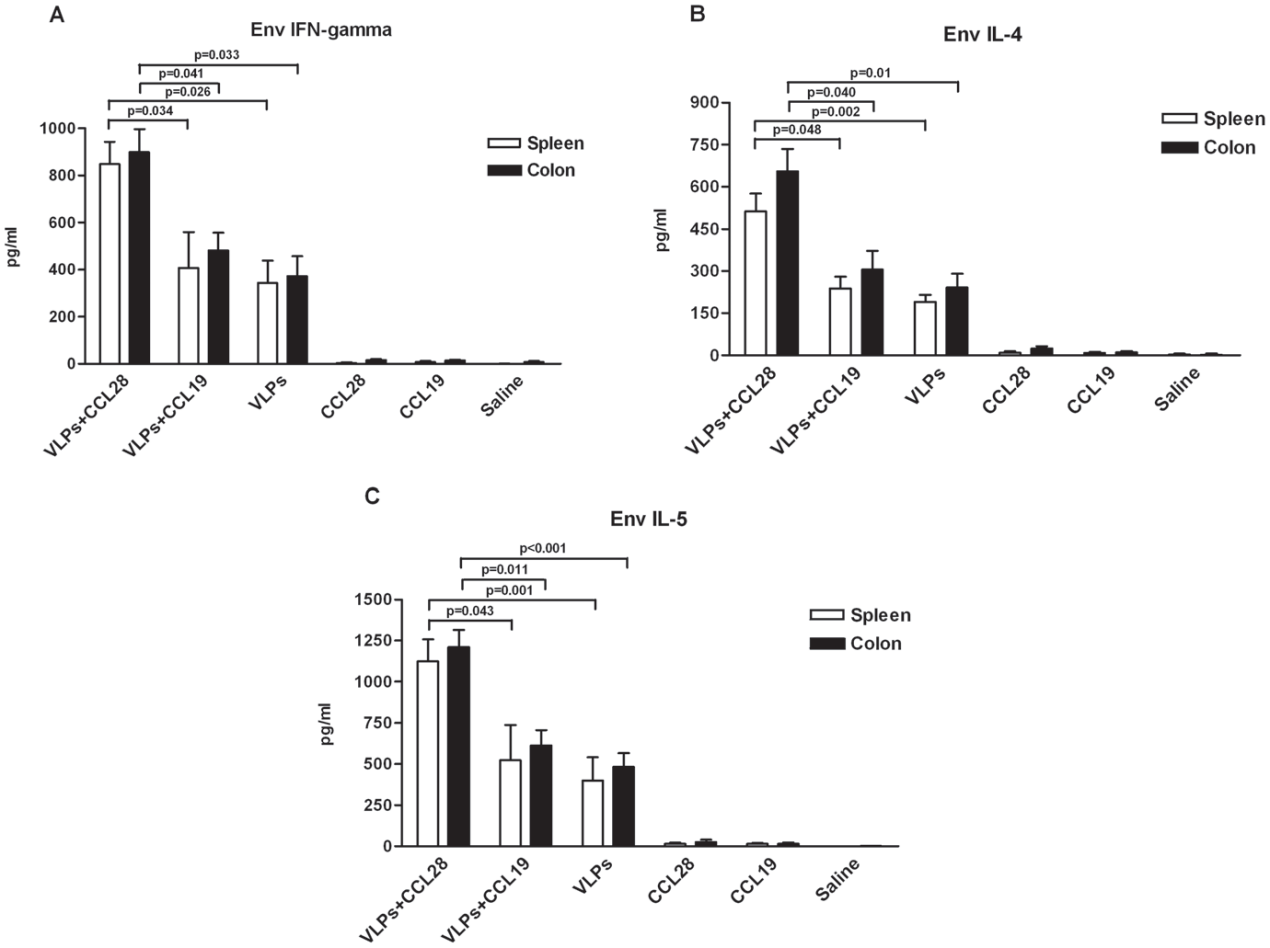
HIV-specific cell-mediated responses. HIV-specific cytokine production in HIV-1-VLP_IIIB_-CCL28, HIV-1-VLP_IIIB_-CCL19, HIV-1-VLP_IIIB_, CCL28 alone, CCL19 alone and control (saline)-treated mice is shown. Panels *A*, *B*, and *C* respectively represent IFN-γ, IL-4 and IL-5 concentrations (pg/ml) in the supernatants from *ex vivo* HIV-1_IIIB_/gp120-restimulated splenic and PP/colonic cells. Mean values (mock subtracted) ± SD and statistically significant differences are represented.

Splenic and colonic cells were restimulated with recombinant HIV-1_IIIB_/Env-gp120 to quantify Th1-type (IFN-γ) and Th2-type (IL-4 and IL-5) cytokines production. Results showed a significant increase of IFN-γ production by both spleen and PP/colonic cells from HIV-VLP_IIIB_-CCL28-treated mice as compared to mice receiving either HIV-VLP_IIIB_-CCL19 (spleen p = 0.034; colon p = 0.041) or VLP alone (spleen p = 0.026; colon p = 0.033) (Fig. 3A). A significant increase of IL-4 and IL-5 production was observed as well in HIV-VLP_IIIB_-CCL28-treated mice compared with either HIV-VLP_IIIB_-CCL19 (spleen p = 0.048 and 0.043; colon p = 0.040 and 0.011) or VLP alone animals (spleen p = 0.002 and 0.001; colon p = 0.01 and <0.001) (Fig. 3B and 3C). In contrast, and as expected, anti-HIV-1/Env-gp120 responses were not observed either in pre-immune mice or in the mice group-treated with CCL28 alone, CCL19 alone, or saline.

### HIV-1/Env gp120-specific systemic humoral response

**Figure 4:**
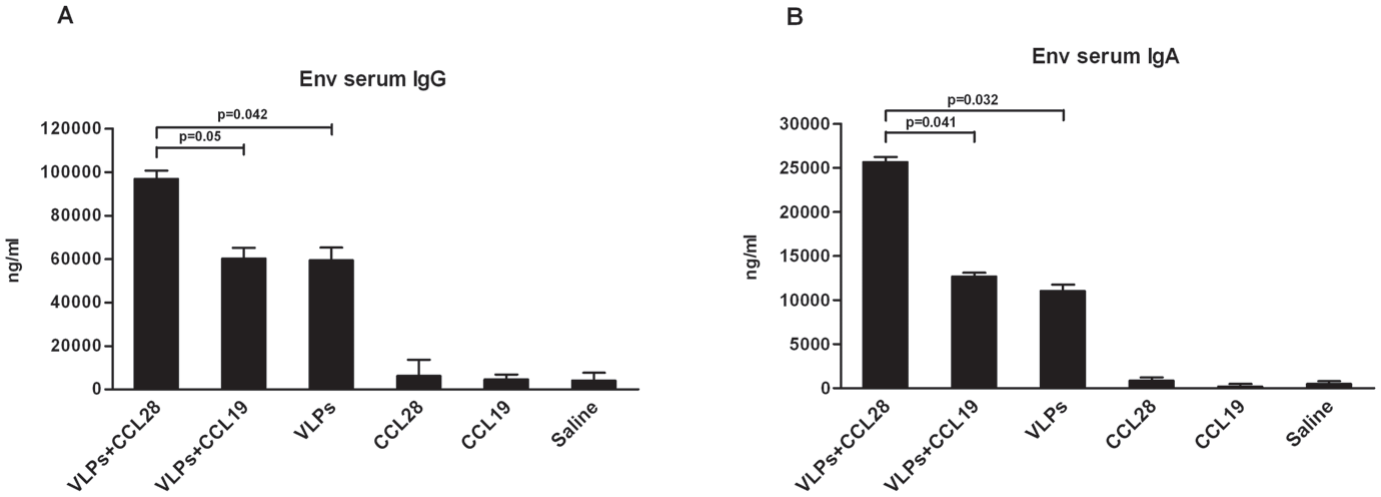
Anti-HIV-1 systemic humoral immunity. The anti-HIV-1 antibody production in mouse sera is shown at days 0 (T0), 14 (T1 and 28 (T2) from each of the following immunized mice groups: HIV-1-VLP_IIIB_-CCL28, HIV-1-VLP_IIIB_-CCL19, HIV-1-VLP_IIIB_, CCL28 alone, CCL19 alone and control (saline)-treated mice. Levels of anti-HIV-1/Env-specific IgG (*A*) and anti-HIV-1/Env-specific IgA (*B*) in sera. The data are expressed as the mean ± SD (n = 5 mice per group) from three independent experiments. Statistically significant differences between groups and the reference HIV-1-VLP_IIIB_-CCL28 group are indicated.

Serum was collected two weeks after the final immunization to measure Env-specific antibodies; ELISA were performed on microwell plates coated with HIV-1_IIIB_ Env gp120. Results showed that serum Env-specific IgG and IgA were increased in HIV-VLP_IIIB_-CCL28 mice compared to HIV-VLP_IIIB_-CCL19 (p = 0.05 and 0.046) or HIV-VLP_IIIB_ mice (p = 0.042 and 0.04) (Fig. 4). Env-specific antibodies were not observed in pre-immune mice, CCL28 alone, CCL19 alone, and saline animals.

### Total and HIV-specific mucosal IgA

**Figure 5:**
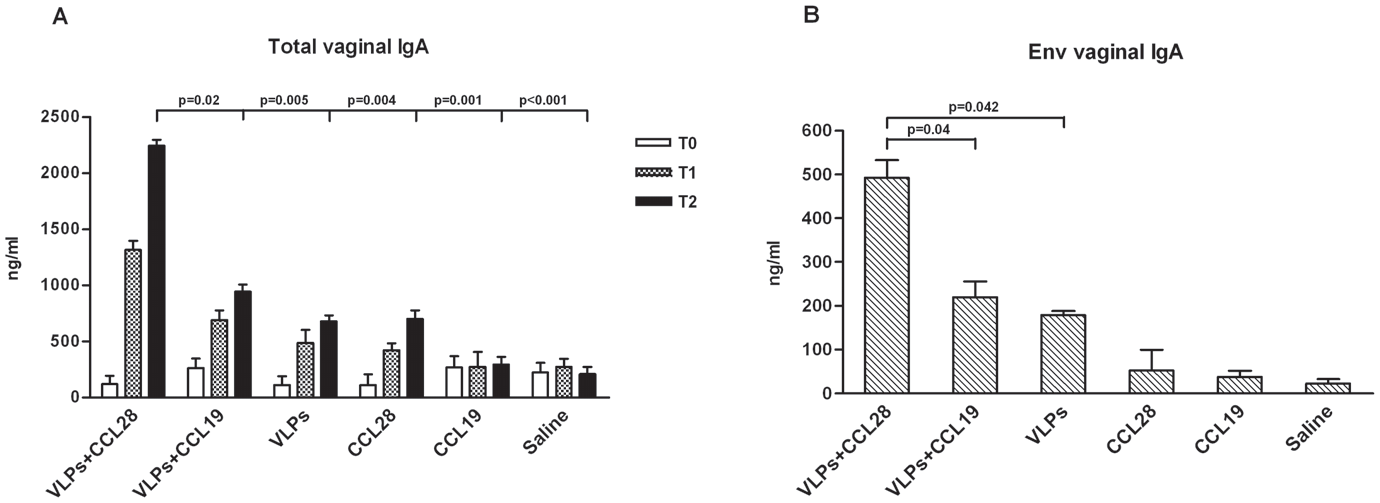
Anti-HIV-1 humoral immunity in mucosal sites. Mucosal anti-HIV-1 antibody production at days 0 (T0), 14 (T1) and 28 (T2) is represented for the following group of immunized mice: HIV-1-VLP_IIIB_-CCL28, HIV-1-VLP_IIIB_-CCL19, HIV-1-VLP_IIIB_, CCL28 alone, CCL19 alone and control (saline)-treated mice. Total (*A*) and anti-Env (*B*) IgA levels present in vaginal secretions. The data represent mean values ± SD (n = 5 mice per group) of three independent experiments. Statistically significant differences compared to the HIV-1-VLP_IIIB_-CCL28 group are represented.

To assess CCL28-induced migration of Ig-ASCs at mucosal sites, both total and Env-specific antibody responses were quantified in vaginal secretions of immunized and control mice. Total IgA levels were significantly augmented in vaginal secretions of HIV-VLP_IIIB_-CCL28-treated mice compared to all other animals (vs. VLP_IIIB_-CCL19 p = 0.04, vs. VLP_IIIB_ alone p = 0.008, vs. CCL28 alone, CCL19 alone, and saline p<0.001,) (Fig. 5A). Notably, Env-specific IgA levels were significantly increased as well in vaginal secretions of HIV-VLP_IIIB_-CCL28 mice compared to HIV-VLP_IIIB_-CCL19 and HIV-VLP_IIIB_ alone animals (p = 0.045 and p = 0.005, respectively) (Fig. 5B).

### Neutralization activity of sera and mucosal secretions

**Figure 6:**
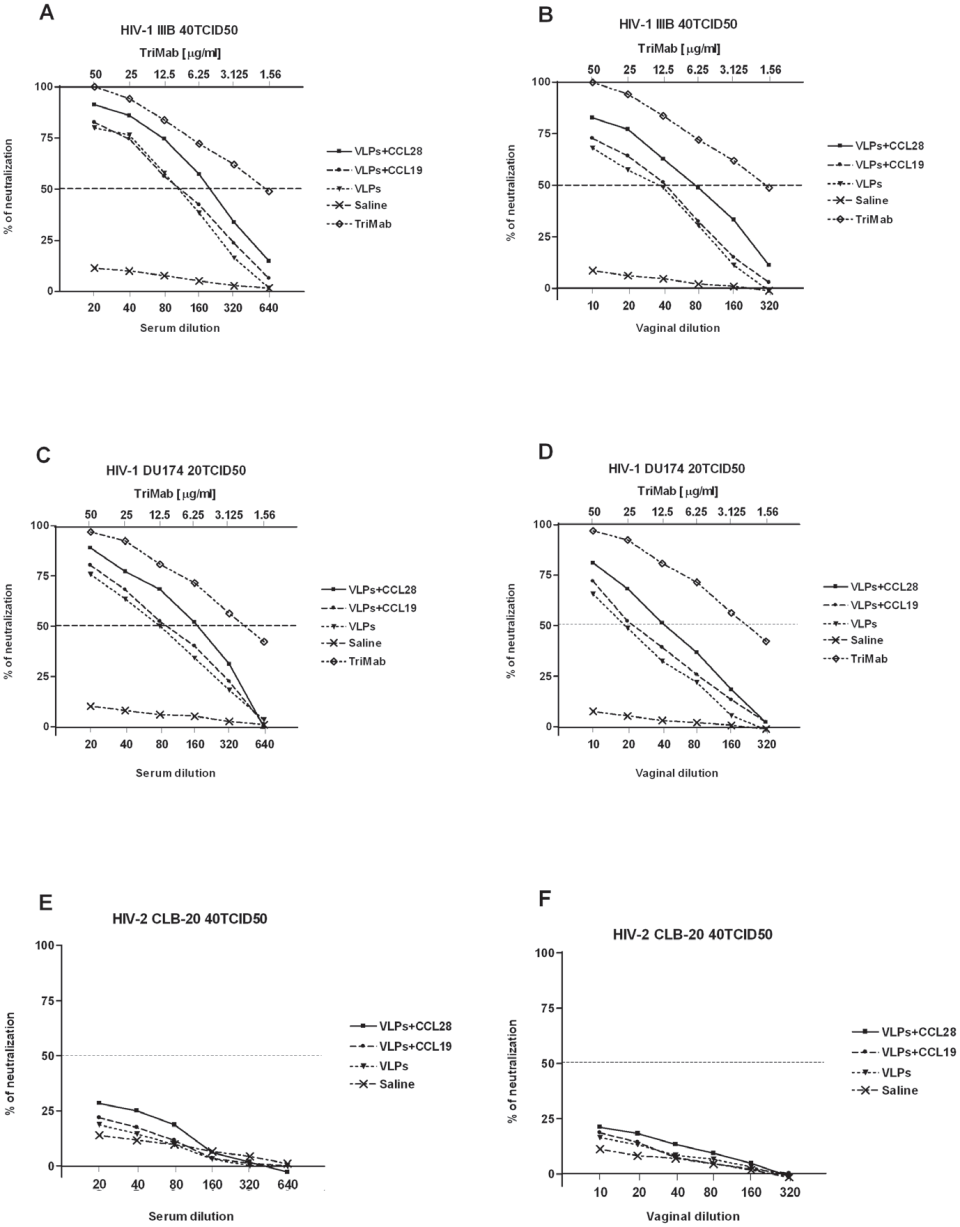
Mouse sero-neutralization activity against HIV-1 or HIV-2. Experiments were run on pooled sera from HIV-1-VLP_IIIB_-CCL28, HIV-1-VLP_IIIB_-CCL19, HIV-1-VLP_IIIB,_ or control (saline)-treated mice. Upper panels show *ex vivo* neutralizing activity against HIV-1_IIIB_ of immune sera (*A*) or vaginal secretions (*B*). Middle panels show *ex vivo* neutralizing activity against HIV-1_DU174_ of immune sera (*C*) or vaginal secretions (*D*). Lower panels show *ex vivo* neutralization activities against HIV-2_CLB-20_, respectively from immune sera and vaginal secretions *(E, F*). The neutralization titer of immune sera and of vaginal secretions is represented as percentages of the virus replication as compared with control samples. Dotted line indicates 50% neutralizing activity. A pool of three neutralizing anti*-*HIV-1 monoclonal antibodies (IgG1b12, 2F5 and 2G12, termed TriMab) was serially diluted at 2-fold dilutions, starting from 50 µg/ml to 1.56 µg/ml, and was used as a positive control in the neutralization assay against HIV-1_IIIB_ and HIV-1_DU174_. Data represent three different lots of independent experiments.

To verify whether the use of CCL28 would result in an enhanced neutralizing activity of Env-specific antibodies, neutralization experiments using pooled sera against HIV-1_IIIB_ and HIV-1_DU174_ were performed. Two different virus strains were used: HIV-1_IIIB_, a subtype B CXCR4-tropic strain, and HIV-1_DU174_, a subtype C CCR5-tropic strain. A marginal (<15%) neutralizing activity was detected in the pre-immune and immune sera of mice treated with either CCL28 alone, CCL19 alone, or saline (data not shown). Immune sera from HIV-VLP_IIIB_-CCL28 treated mice showed a neutralization activity titer of 220 and 160 (50% neutralization activity) against, respectively, HIV-1_IIIB_ at a TCID50 of 40 and HIV-1_DU174_ at a TCID50 of 20 (Fig. 6A and 6C). Conversely, immune sera from HIV-VLP_IIIB_-CCL19 or VLP_IIIB_ alone mice showed a neutralization activity titer of 100 against HIV-1_IIIB_ and of 80 against HIV-1_DU174_ at the same TCID50 of 40 and 20, respectively (Fig. 6A and 6C).

Immune vaginal secretions from HIV-VLP_IIIB_-CCL28-receiving mice showed a neutralization titer of 80 and 40 against, respectively, HIV-1_IIIB_ and HIV-1_DU174_ (Fig. 6B and 6D). Also in this case the neutralization activity against both strains was lower in mice treated with HIV-VLP_IIIB_-CCL19 or VLP_IIIB_ alone mice, resulting in a neutralizing titer of 46 and 18, respectively. No neutralization activity was present when HIV-1_IIIB_ and HIV-1_DU174_ were used at a double TCID50 (data not shown).

The neutralization observed against both a clade B and a clade C virus with vaginal secretions is apparently puzzling considering that antibody concentration in vaginal secretions is lower than in serum. A technical issue with VLP-based vaccines is their tendency to elicit anti-cell antibodies against non-Env membrane proteins that could interfere with the neutralization assays [23]. Hence in traditional assays, such as the PBMC-based neutralization assay, controls are used to identify possible non-specific activity. We employed a HIV-2_CLB-20_ neutralization assay as a control to distinguish anti-Env activity from non-specific effects. Neutralization activity of murine samples against HIV-2_CLB-20_ was marginal (<25%) suggesting that both sera and vaginal secretions of immunized mice are indeed endowed with anti-Env reactivity (Fig. 6E and 6F).

**Figure 7:**
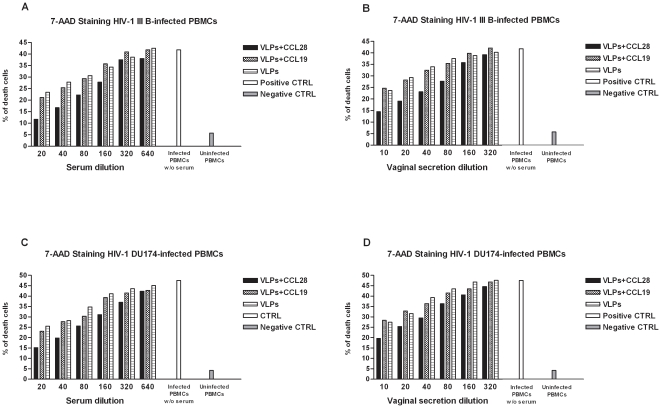
Cell viability. Experiments were run on pooled sera of HIV-1-VLP_IIIB_-CCL28, HIV-VLP_IIIB_-CCL19 and HIV-VLP_IIIB_-treated mice. Upper panels show the percentage of cell death in HIV-1_IIIB_-infected PBMCs mixed with 2-fold serial dilutions of murine sera (*A*) or vaginal washes (*B*) 7 days post-infection. Lower panels show the percentage of cell death in HIV-1_DU174_-infected PBMCs mixed with 2-fold dilutions of murine sera (*C*) or vaginal washes (*D*) 7 days post-infection. Cell survival was measured by 7-AAD staining and flow cytometry analysis. HIV-1_IIIB_ or HIV-1_DU174_-infected PBMCs and untreated uninfected PBMCs were used, respectively, as positive and negative controls. Data represents three different lots of independent experiments.

Furthermore, to verify whether the results could be a consequence of changes in cell viability, HIV-1_IIIB_- and HIV-1_DU174_-infected PBMCs that were either untreated or mixed with matched serum and vaginal secretions were stained with 7-AAD. As shown in Fig. 7, the viability of HIV-1_IIIB_- and HIV-1_DU174_-infected PBMCs was not significantly affected by incubation with either murine sera or vaginal secretions, as the presence of apoptotic and/or necrotic cells could be related to the direct cytopathic effect of the viruses. Of note, the small amount of cell death observed in HIV-VLP_IIIB_-CCL28 treated mice was below the background level of apoptosis detected in tissue culture as indicated by the levels of viability observed in untreated uninfected PBMCs.

**Figure 8:**
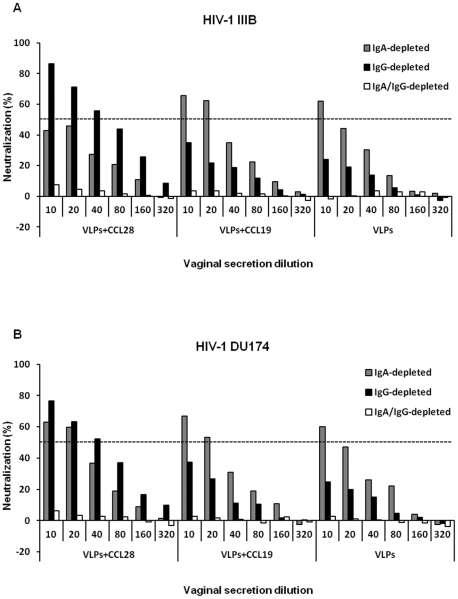
HIV-1 neutralization of Ig-depleted mucosal secretions. Experiments were run on pooled IgG- or IgA- or IgG/IgA-depleted vaginal secretions from HIV-1-VLP_IIIB_-CCL28, HIV-1-VLP_IIIB_-CCL19, HIV-1-VLP_IIIB_ mice. Upper panel show *ex vivo* neutralizing activity against HIV-1_IIIB_ of immune vaginal secretions (*A*). Lower panels shows *ex vivo* neutralizing activity against HIV-1_DU174_ of immune vaginal secretions (*B*). The neutralization titer is represented as percentages of the virus replication as compared with control samples. Dotted line indicates 50% neutralizing activity. The data represent the results of two duplicate-sample assays.

Finally, in order to determine the relative contribution of the main antibody isotypes to the neutralizing activity of vaginal secretions, IgA, IgG or IgA + IgG immune absorption experiments were performed. Results clearly showed that IgA antibodies from VLP-immunized mice played a major role in the neutralization of both HIV-1_IIIB_ and HIV-1_DU174_ and that IgA-mediated neutralization activity was enhanced in a CCL28-dependent manner. In the absence of CCL28, but in the presence of VLP alone or in combination with CCL19, the neutralization level was lower and mostly IgG-dependent. Nevertheless, this was confirmed by the observation that, along with depletion of both IgA and IgG antibodies, the neutralization activity was almost abolished (Fig. 8). Results demonstrated the significant IgA contribution to the observed non-IgG-mediated neutralization of both HIV-1 clades, although not completely excluding the possible minor roles of other Ig subclasses.

### IgA^+^ plasma cells in the gastro-intestinal mucosal lamina propria

**Figure 9:**
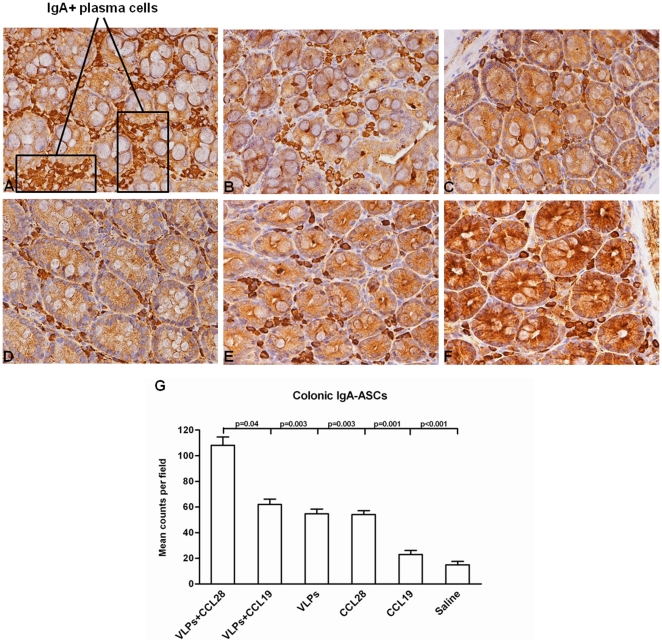
CCL28 effect on colon IgA-ASCs. IgA^+^ plasma cell distribution at the mucosal level in BALB/c mice immunized with HIV-1-VLP_IIIB_-CCL28 (*A*), HIV-1-VLP_IIIB_-CCL19 (B), HIV-1-VLP_IIIB_ alone (*C*), CCL28 alone (*D*), CCL19 alone (*E*) and saline (*F*) is shown. Representative results obtained in the lamina propria of colonic mucosa (2-cm specimens from the anus toward the left colon) are presented. Quantitation of IgA-ASCs in colon specimens (*G*). The sum of 12 high-power fields was calculated for each mouse and data are expressed as means of this sum ± SD for each group.

Since the GI MLP is the principal site of HIV replication during primary infection the induction of virus-specific- mucosal immunity would be beneficial as it could obstacle the establishment of infection. As the results herein demonstrated that the up-regulation of CCL28-CCR3/CCR10 circuit is correlated with increased concentrations of both systemic and mucosal HIV-specific IgA, we analyzed IgA-ASC distribution in the GI mucosal *lamina propria*. IgA^+^ plasma cells were clearly identified by immunopositive staining in the cytoplasm of cells with plasma cell-like morphology. Results showed the presence of numerous IgA^+^ cells clustered within the *lamina propria* of the colonic mucosa in HIV-VLP_IIIB_-CCL28 mice. The number of IgA^+^ plasma cells observed in the colonic MLP of these mice was significantly higher compared to all the other groups of mice (p<0.001), in whom only rare and isolated IgA^+^ cells could be identified. Representative results are shown in Fig. 9. The higher frequency of IgA-ASCs in the gastrointestinal mucosa of HIV-1-VLP_IIIB-_CCL28 mice group as compared with the CCL28 alone group is not surprising, as it could be directly related to the induction of antigen-specific humoral immunity. Exposure to HIV-1_IIIB_ VLPs indeed leads to activation of naïve B-cell clones specific for HIV-1 antigens that then undergo clonal expansion, mitotically forming large populations of cells including memory cells and antibody-secreting plasma cells. Thus administration of the murine CCL28 gene-expressing vector to HIV-1-VLP_IIIB_ mice would result in a large influx of both polyclonal and antigen-specific IgA-ASCs to the mucosa.

## Discussion

The vast majority of newly acquired HIV infections are sexually transmitted. In this modality of infection the virus initially targets the vaginal or rectal mucosa; preventative vaccines or microbicides should thus be designed to protect such mucosae [24], [25]. With the exception of the moderately promising results of the RV144 trial (the so called “Thai trial”) [26], [27], [28], no candidate HIV-1 vaccine has shown convincing successful results. Alternate approaches based on the prevention of HIV infection through the use of topical microbicides have also exhibited unsatisfactory results [29], even if very recently promising breakthrough results were presented [30]. Therefore, strategies to protect mucosal tissues, including vaccines designed to play protective and therapeutic roles *via* the elicitation of systemic immune responses, are needed. To better understand how to construct a mucosal vaccine that could protect the tissues that are the earlier targets of HIV infection, we focused on CCL28, a chemokine that plays an important role in the migration of IgA-ASC in the mucosal lamina propria [7].

Results presented herein demonstrate that immunization of mice with Env-expressing VLPs in the presence of CCL28 results in the modulation of the whole CCL28-CCR3/CCR10 circuit and optimizes immunization-induced HIV-specific immune response. Thus, cytokine secretion by spleen and colon cells, as well as total and HIV-specific IgA and IgA^+^ plasma cells were significantly increased in the presence of CCL28. Notably, HIV-specific IgA and IgG titers were significantly increased in serum as well, suggesting that, systemic as well as mucosal immune responses are efficiently modulated by CCL28. Finally, the observation that the neutralization ability of serum and vaginal washes of immunized mice in the presence of CCL28 was increased indicates that the immune modulation induced by this chemokine is associated with an augmented capacity to down-modulate HIV infectivity.

This study shows that the objective of up-regulating potentially beneficial mucosal immune responses is achievable, at least in the mouse model, by CCL28. Our results demonstrate that CCL28 indeed results in the recruitment of IgA-ASCs in mucosal epithelial tissues and this effect is associated with an increased neutralization activity. The increased neutralization ability toward at least two different HIV clades exhibiting the two major X4 and R5 tropisms, detected in immune sera and vaginal secretions, indicates that the reduction of HIV infectivity *in vivo* could be achieved by such adjuvant.

The tissue-targeted lymphocyte migration is strongly regulated by a complex network of chemokines [9]. CCL28 has been shown to govern the ASC homing, and in particular that of IgA-ASCs to gastro-intestinal tissues, upper aero-digestive and mammary glands [31]. CCL28 binds to CCR3 and CCR10 and CCR10 is considered to be a unifying chemokine receptor playing a pivotal role in plasma blasts homing of extra-intestinal effector sites [8], [32]. The ability of the CCL28-CCR3/CCR10 circuit to chemoattract ASCs into multiple mucosal sites has indeed been a founding element of the concept of a common mucosal immune system. Recent results indicate that CCL28 has a broad and unifying role in the IgA physiology of the mucosal immune system [33], [34].

IgA are mostly mucosal antibodies involved in the first line of defense of adaptive immunity facing pathogens. The anti-HIV-1 mucosal IgA are not only observed in HIV-1 infected individuals [35], [36], but also have been detected in some, and not in all HIV-exposed seronegative individuals (HESN) [37], [38], [39]. IgA isolated from cervico-vaginal secretions of these individuals are capable of inhibiting virus transcytosis through epithelial layers *in vitro* and have a potent neutralizing activity [40], [41], [42]. Because IgA from HESN seem to contribute to the prevention of infection of these individuals, it is reasonable to infer that vaccine procedures capable of eliciting a massive IgA response would be beneficial by contributing to the containment of HIV-1 infection.

The gut-associated lymphoid tissue (GALT) contains the majority of T-cells in the body; recent data showed that GALT is the preferential target for HIV replication during the acute phase of HIV infection [43], [44]. These observations have strengthened the need for mucosal vaccine for the prevention of HIV infection [45], [46]. Results presented here indicate that CCL28 significantly increases the quantity of mucosal ASCs in the colon as well. Although in this study we did not directly measured rectal IgA, it is plausible that increased quantity of tissue IgA-producing ASCs would results in an increased amount of secreted antibodies, thus the situation of IgA in rectal washes would parallel what is observed in vaginal washes of CCL28-receiving mice.

In this study we report that intramuscular administration of HIV-1_IIIB_-VLPs in the presence of the CCL28-expressing plasmid correlates with a robust up-regulation of chemokine receptor expression on circulating B-cells and an increase in both systemic and mucosal immunity. This could apparently be surprising, as parenteral immunization is generally thought not to induce significant immune responses at mucosal surfaces. However, recent studies have revealed the possibility of cross-talk between systemic compartments and mucosal tissues [47], [48], [49]. Furthermore, CCL28 was shown to be constitutively expressed by bone marrow stromal cells, suggesting a role for this chemokine in the integration between the mucosal and the systemic immune responses through interaction with CCR10^+^/CCR3^+^ circulating B-cells [7]. It is tempting to speculate that the enhanced expression of CCL28 in the draining lymph nodes following vaccination would upregulate CCR3 and CCR10 in locally recruited CD19^+^/CCR3^+^ and CD19^+^/CCR10^+^ cells. Activated antigen-specific B-cells generated in draining lymph nodes could disseminate to GALT, as CCR3 and CCR10 would allow their trafficking to mucosal tissue sites that constitutively express CCL28. Following homing to GALT, activated lymphocytes would undergo preferential isotype switching to IgA under the influence of cytokines such as IL-4, IL-5 or IL-10. This hypothesis is supported by the observation that Th2 cytokine responses were augmented in the GALT of mice treated with HIV-1_IIIB_-VLPs in the presence of CCL28. Alternatively, CCR10^+^ dendritic cells capable of presenting HIV-specific peptides could traffic from draining lymph nodes to Peyer's patches and mesenteric lymph nodes and initiate the production of antigen-specific IgA-ASCs that would subsequently home to the *lamina propria* of GALT. It is conceivable that CCL28 may function not only as chemoattractant but also as a co-activator, as the level of systemic antibody and cytokine would not be greatly affected, although the mechanisms underlying the induction of antigen-specific cellular and humoral responses at both the systemic and mucosal level needs to be further investigated.

As far as the CCL28-CCR3/CCR10 circuit is concerned, the percentage of CD19^+^/CCR3^+^ cells was unexpectedly high as compared with previous results observed in humans [12]. To date, CCR3 expression on B-cells and the biological functions of the CCR3 ligand CCL28 for murine B-cells have not been characterized. In humans, besides being expressed in eosinophils, basophils and dendritic cells [50], CCR3 has been shown to be selectively expressed in T helper 2 (Th2) cells [51] and in antibody-secreting plasma blasts and plasma cells [6], [7]. If this observation holds true also for the mouse system, the expression of CCL28 in the lymph nodes draining the site of vaccine inoculation would result in a more efficient recruitment of CD19^+^/CCR3^+^ cells, providing a plausible basis for B-cell activation and clonal expansion upon encounter with vaccine antigens. Subsequently, lymphocyte recirculation between tissues and blood compartments would account for the high frequency of CD19^+^/CCR3^+^ splenocytes observed in the mouse model. However, it should also be taken into account the source of B-cells used for the flow cytometric analyses, as CCR3 has been shown to be virtually absent on freshly isolated human peripheral B-cells [52], while it has been detected in mouse spleen and liver [53], [54], [55]. Nevertheless, further work needs to be done to establish the expression of CCR3 on murine B-cells and the role of this receptor in the CCL28-mediated lymphocyte homing.

It is noteworthy that results showed significant differences in the plasmid-induced serum levels between CCL28 and CCL19 *in vivo,* although pCCL28 and pCCL19 contained the same murine Cytomegalovirus (CMV) promoter and no substantial differences were observed in the *in vitro* production of CCL28 and CCL19 by HEK/293T-cells (data not shown). However, taking into account the physiological variation in normal serum levels between these two chemokines in the mouse model, only the increase in CCL28 but not in CCL19 production from baseline resulted in an enhanced immune response in HIV-1-VLP_IIIB_ treated mice after administration of the chemokine expression plasmid. Taken together our data allow us to speculate that the higher adjuvant efficacy of pCCL28 than pCCL19 is not related to the variability in the plasmid-induced chemokine production but to the different mechanism of action of the encoded chemokine, CCL28 recruiting IgA-ASCs to mucosal effector sites and CCL19 directing T-cell and DC migration to lymphoid organ T zones.

In conclusion, we show that CCL28 used as an adjuvant has a robust immunomodulatory effect on potentially beneficial mucosal and systemic immune responses. Notably, the beneficial immunomodulatory effects of CCL28 is not likely limited to HIV infection as this chemokine was recently shown to be effective in eliciting long-lived antibody responses that neutralized influenza A/PR8/34 and protected mice from morbidity and mortality associated with a lethal intranasal viral challenge [22]. These findings suggest that CCL28 could play a useful role in increasing the efficiency of preventive vaccines for HIV infection.

## Materials and Methods

### Production of HIV-1_IIIB_ VLP

HIV-1 virus like particles (VLP) were produced by transient transfection of HEK/293T-cells (human embryo kidney carcinoma cell-line, T clone) (Sigma-Aldrich, Germany) with the plasmids pCD-Hgpsyn and pConBgp160-opt. pCD-Hgpsyn containing the codon-optimized gag-pol sequence of HIV-1_IIIB_ kindly provided by Geneart (Regensburg, Germany). pConBgp160-opt encodes a consensus full-length HIV-1/subtype B (HIV-1/B) envelope obtained from the AIDS Research and Reference Reagent Program, Division of AIDS, NIH, (Dr. Beatrice Hahn). Confluence of 80–90%, HEK/293T cells were transfected with 40 µg of each plasmid in DMEM medium supplemented with PEI [10 mg/ml], 5% FCS and penicillin/streptomycin. The transfection medium was changed 18 hr after transfection with DMEM medium supplemented with 2% FCS and penicillin/streptomycin and harvested 48 hr later. The harvested medium was centrifuged at 300×g for 10 min and filtered through a 0.45 µm filter to remove cell debris. VLP were further purified and concentrated from the conditioned medium by ultracentrifugation through a 20% sucrose gradient and a subsequent ultracentrifugation at 28,000 rpm for 2 hr a SW28 rotor (Beckman, CA). Supernatants were discarded and the pellets containing viral particles were resuspended in PBS. The endotoxin level was measured by QCL-1000^®^ Chromogenic LAL Endpoint Assay (Cambrex, Germany) according to the manufacturer's instructions. The concentration of viral proteins in the final VLP preparation was determined by an in-house ELISA and corresponded to 1.5 µg/ml of HIV-1 Env and 0.55 µg/ml of HIV-1 Gag. The calibration curve was set up by using recombinant HIV-1_IIIB_ Env gp120 (Baculovirus) (EVA607 supplied by the NIBSC centralized facility for AIDS Reagents supported by EVA EU Program) and Gag^pr55^ (AIDS Research and Reference Reagent Program, Division of AIDS, NIAID, NIH).

### Production of chemokine-expressing plasmids

The MEC/CCL28 murine chemokine gene was digested with AgeI from the pORF-mCCL28 plasmid (InvivoGen, San Diego, CA) and a blunt end was produced with T4-DNA polymerase. Following digestion with NheI, the resulting 410 bp fragment was inserted into a pCpG expression vector, previously digested with ScaI/NheI. The CCL19/MIP-3β murine gene from the pORF5-mMIP3β inserted into a pCpG expression vector (InvivoGen, San Diego, CA) as described above was used as a negative control. The endotoxin level was measured by QCL-1000^®^ Chromogenic LAL Endpoint Assay (Cambrex, Germany) according to the manufacturer's instructions.

### Immunization procedures

The study was approved by the Italian Ministry of Health (Permit Number: 02/010) and animals were managed according to the principles of the "Guide for the Care and Use of Laboratory Animals" and in accordance with the Italian national law (Legislative Decree. 116/1992) and the recommendations of the European Community (86/609/CEE) for the care and use of laboratory animals. Adult inbred female Balb/c mice, 6–8 weeks old, were purchased from Charles River Laboratories (Calco, Italy). Mouse colonies were maintained on a 12-h light-dark cycle in cages of 5 animals with water and food provided *ad libitum*. Mice were randomized in six groups (5 mice/group) to receive: HIV-1_IIIB_ VLP (150 ng Env/mouse) in combination with the murine CCL28 gene inserted into a CpG-free expressing plasmid (pCCL28) (50 µg/mouse) or HIV-1_IIIB_ VLP in combination with the murine CCL19 gene also inserted into a CpG-free expressing plasmid (pCCL19) (50 µg/mouse) or HIV-1_IIIB_ VLP alone (150 ng Env/mouse) or pCCL28 alone (50 µg/mouse) or pCCL19 alone (50 µg/mouse). Control mice were treated with endotoxin-free phosphate-buffered saline (PBS, Organon Teknika Corp., Durham, NC). HIV-1_IIIB_ VLP, pCCL28 and pCCL19 were administrated intramuscularly in quadriceps muscles. Mice were immunized on day 0, boosted on day 14 and sacrificed by cervical dislocation on day 28.

### Blood and vaginal secretion collection

Fifty microliters of blood were collected by tail vein bleeding and added to 100 µl of phosphate-buffered saline (PBS, Organon Teknika Corp.) containing 100 IU of heparin (Hip-PBS, Sigma). Serum was obtained after centrifugation at 800 g for 10 min and stored at -80°C. Vaginal secretions were collected with 50 µl of pre-warmed PBS supplemented with 100X-concentrated protease inhibitor cocktail (1%, v/v) (O-complete, Roche Applied Science, Germany) by repeated aspiration until a discrete quantity of mucus was recovered. Material was collected and centrifuged at 12,000 g for 10 min, the supernatant was transferred to sterile microcentrifuge tubes and frozen at −20°C until use. The endotoxin level was measured by QCL-1000^®^ Chromogenic LAL Endpoint Assay (Cambrex, Germany) in both sera and vaginal secretions according to the manufacturer's instructions, resulting in a an endotoxin concentration of 0.8 ng/ml and 0.4 ng/ml, respectively.

### Tissue harvesting

Mice were anesthetized for 2 min with ether gas and sacrificed by neck dislocation. Spleens were excised under sterile conditions in a laminar flow hood and put through a 100 µm plastic strainer (BD Falcon 2350, BD Biosciences, Bedford, MA) for cell recovery. Splenocytes were layered on a continuous 40–100% Percoll gradient (Sigma) and washed twice in PBS to obtain lymphocyte-rich cells. Cell viability was determined using Trypan blue staining. Splenocytes were resuspended in cell culture medium (RPMI 1640) (Organon Teknika Corp.) and used in cell culture assays. Peyer's patches (PP)/colonic mononuclear cells were recovered from freshly obtained specimens. Colon specimens were first washed in HBSS-with Phenol Red (BioWhittaker Inc., Walkersville, MD) cut into 0.5-cm pieces. They were then incubated twice, each time for 15 min in 0.37 mg/ml EDTA-HBSS (Sigma) and 0.145 mg/ml dithiothreitol (Thermo Fisher Scientific, Rockford, IL) at 37°C. Tissues were further digested at 37°C for 10 min with 400 U/ml of Collagenase D (Roche) and 0.01 mg/ml of DNase I (Sigma). Tissue-released cells were suspended in a continuous 100%–40% Percoll gradient to obtain the lymphocyte-enriched population. Mice recta were also analyzed by immune-histochemistry.

### Cell count

Cell count was performed with the automated cell counter ADAM-MC (Digital Bio, NanoEnTek Inc, Korea). ADAM-MC automatic cell counter measures total cell numbers and cell viabilities by cutting-edge detection technologies. In addition to Trypan blue staining, ADAM-MC procedure was carried out using two sensitive fluorescence dye staining solutions, AccuStain Solution T (Propidium Iodide/lysis solution) and AccuStain Solution N (Propidium IodideI/PBS). AccuStain Solution T permeabilizes plasma membrane stain nucleus that allow measurements of total cell enumeration, while AccuStain Solution N exclusively stains non-viable cells. A 532 nm optic laser is automatically focused onto the cell suspension contained into a disposable microchip where cell analysis is made with a CDD camera.

### CCL28 and CCL19 expression *in vivo*

*In vivo* expression of active CCL28 and CCL19 chemokines in serum samples encoded by the plasmids was verified using commercial ELISA kits (R&D Systems, Minneapolis, MN) and following the manufacturers' instructions. Chemokine concentration was calculated from a standard curve obtained with the corresponding recombinant mouse chemokine.

### Flow cytometry analysis of CCR3 or CCR10 expression

CCR3 or CCR10 receptor expression was evaluated on splenocytes. Cells were resuspended in PBS and their surface stained with mAbs CD3e PE- Cy5 (Armenian Hamster IgG isotype, eBioscience, San Diego, CA), anti-mouse CD14 PE-C-conjugated Cy5 (rat IgG2a isotype, eBioscience), PE-Cy5-conjugated anti-mouse CD19 (rat IgG2a isotype, eBioscience), a rat FITC-conjugated anti-mouse CCR3 IgG2a isotype (R&D Systems, Minneapolis, MN), and a rat anti-mouse CCR10 PE IgG2b isotype (R&D Systems). Following incubation 15 min at room temperature in the dark, cells were washed 3 times in PBS and fixed in 1% formaldehyde. All the cytometric analyses were performed using an FC500 flow cytometer (Beckman-Coulter, Miami, FL) equipped with a double 15-mW argon ion laser operating at 456 and 488 interfaced with an Intercorp (Venice, Italy) computer. For each analysis 20,000 events were acquired and gated on CD3 or CD14 or CD19 expression and side scatter properties. Green fluorescence from FITC (FL1) was collected through a 525-nm band-pass filter, orangered fluorescence from R-PE (FL2) was collected through a 575-nm bandpass filter and red fluorescence from Cy5PE (FL4) was collected through a 670-nm band-pass filter. Data were collected using linear amplifiers for forward and side scatter and logarithmic amplifiers for FL1, FL2, FL4, and FL5.

### Cytokine assays

IFN-γ, IL-4 and IL-5 production were measured in the supernatants from splenocytes as well as in PP/colonic T cells after *ex vivo* re-stimulation with recombinant HIV-1_IIIB_/Env gp120 (NIBSC) using commercial ELISA kits (R&D Systems) and following manufacturers' procedures.

### Evaluation of humoral immune responses

Anti-HIV-IgG and IgA were measured in sera and vaginal secretions by an ELISA method based on a recombinant HIV-1 envelope protein. 96 well ELISA plates were coated overnight at 4°C with 100 µl of 4 µg/ml recombinant HIV-1_IIIB_ Env gp120 (Baculo) (NIBSC). Plates were washed three times with PBS-Tween-20 0.05% buffer, and incubated for 2 hr at 37°C, 5% CO_2_ with PBS containing 3% of Bovine Serum Albumin (BSA) (Sigma, St Louis, MO) to block non-specific protein binding sites. Serum dilutions 1/10 to 1/10000 and vaginal wash dilutions 1/2 to 1/1000 were incubated for 2 hr at 37°C, 5% CO_2_. Plates were then washed three times and a goat HRP-conjugated anti- α-chain IgA antibody mouse (Sigma) or a goat HRP-conjugated anti-mouse IgG antibody (Jackson Immuno-Research, West Grove, PA) diluted 1∶1000 or 1∶30000 in PBS/BSA respectively was added to the plates. Following 1 h of incubation at 37°C, 5% CO_2_, the plates were washed and incubated with tetramethylbenzidine (TMB, R&D Systems) substrate solution for 30 min at RT. The addition H_2_SO_4_ 1.8 M stopped the colour reaction. IgG or S-IgA concentrations were measured at an Optical density absorbance λ = OD_490_. Total IgA from vaginal secretions were measured by ELISA as described above, excepting the coating that was carried out at 4°C overnight with 100 µl of a goat anti-mouse IgA antibody (Kamiya Biomedical Company, Seattle, WA). Sample concentrations were determined from standard curves, using purified Ig standard mouse IgA and IgG (Sigma) assayed in parallel; values were expressed in nanograms per milliliter.

### Ig depletion of vaginal secretions

Streptococcal protein G and Staphilococcal protein A were used to specifically remove IgG and/or IgA from mucosal samples. Mouse vaginal secretions were diluted 1∶2 with RPMI 1640 medium (Organon Teknika Corp.) supplemented with 1% penicillin/streptomycin and incubated with an equal volume of Sepharose-immobilized recombinant protein G (PGS) and/or Sepharose-immobilized recombinant protein A (PAS) (Sigma Chemical Co., St. Louis, MO). The incubation was carried out for 2 h at 37°C with constant gentle rotation to prevent sedimentation of the PGS and/or the PAS. After centrifugation for 2 min at 1000 g, the supernatant was withdrawn and incubated for 12 h on the rotator at 4°C with a second aliquot of PGS and/or PAS equal to the first. The specimens were then returned to the 37°C rotating incubator for an additional 2 h. After centrifugation at 1000g for 2 min, the 1∶2 diluted vaginal washes, either IgG-depleted or IgA-depleted or IgG/IgA-depleted, were harvested.

### HIV-1 neutralization assay

HIV-1 neutralization assay was performed in a pooled donor PBMC-based assay. Briefly, pooled sera from pre-immunized and immunized mice were complement-depleted by heat inactivation at 56°C for 60 min and serially diluted at 2-fold dilutions, starting from 1/20. Pooled vaginal secretions were serially diluted at 2-fold dilutions, starting from 1/10. Vaginal secretions were assayed before and after Ig depletion. Sera and vaginal secretions were incubated in a humidified 5% CO_2_ incubator at 37°C for 60 min in duplicate in 96-well plates with the laboratory strain HIV-1_IIIB_ and the primary isolate HIV-1_DU174_ (NIBSC) of 40 and 20 TCID50, respectively. One hundred thousand PBMCs pooled from HIV-negative donors were stimulated with PHA for 48 hr, added to each well, and incubated overnight (18 hr) at 37°C. Cells were extensively washed with RPMI 1640 (Organon Teknika Corp.), centrifuged at 400 g for 10 min and fresh medium (RPMI 1640, 200 mM L-glutamine, 20% fetal calf serum, 10 U IL-2, 1% penicillin and 2% streptomycin) was replaced on day 1 and day 3. After 7 days, supernatants were harvested and p24 antigen levels were determined by ELISA according to the manufacturer's instructions (Perkin-Elmer, Waltham, MA). Five hundred thousand uninfected and infected PBMCs either not treated or mixed with matched serum and vaginal secretion dilutions from immunized mice were also stained with 7-AAD viability dye (Beckman Coulter) to test cell toxicity of murine samples. After 20 min incubation at room temperature, cells were centrifuged at 800 g for 10 min in PBS and analyzed by flow cytometry. HIV-2_CLB-20_ (NIBSC) neutralization assay was performed in a PBMC-based assay to identify non-specific activity of murine samples and the protein p27 was detected by ELISA according to the manufacturer's instructions (ZeptoMetrix Corp., NY).

### Immunohistochemistry analyses

Tissues obtained from the colon of mice (2-cm specimens from the anus toward the left colon) were fixed in 10% buffered-formalin for 24 hr at room temperature and embedded in paraffin. Haematoxylin-eosin stained sections were used for histological evaluation. The evaluation of IgA^+^ plasma cells was made on 3 µm paraffin embedded slides, the sections were dewaxed in xylene, rehydrated in an ascendent ethanol scale and pre-treated in a microwave oven (two cycles for 5 minutes each at 780 W, in EDTA buffer, pH 8). Endogen biotin and non-specific signals were blocked with appropriated reagents. For immuno-histochemistry, a goat anti-mouse IgA (dilution 1∶400, AbD Serotec) was used, slides were incubated for 2 hr at room temperature in a humid chamber, washed in PBS, and revealed by biotinylated anti-goat IgG (dilution 1∶50, 1 hr incubation, R&D Systems, London, UK) followed by HRP-Conjugated Streptavidin (30 min incubation, R&D Systems, London, UK). The chromogen was 3, 3′-diaminobenzidine free base (DAB).

### Statistical analysis

Comparisons between groups were analyzed to evaluate immunological differences. Kruskaal-Wallis analysis of variance was performed for each variable; Bonferroni correction was applied to the results. Two-sided p-values were considered. Data analysis was performed using the SPSS statistical package (SPSS Inc. Chicago, IL).
